# Geological methane emissions and wildfire risk in the degraded permafrost area of the Xiao Xing’an Mountains, China

**DOI:** 10.1038/s41598-020-78170-z

**Published:** 2020-12-04

**Authors:** Wei Shan, Zhichao Xu, Ying Guo, Chengcheng Zhang, Zhaoguang Hu, Yuzhuo Wang

**Affiliations:** 1 Institute of Cold Regions Science and Engineering, Northeast Forestry University, Harbin, 150040 China; 2 Field Scientific Observation and Research Station of the Ministry of Education - Geological Environment System of Permafrost Area in Northeast China(FSSE-PFNEC), Harbin, 150040 China

**Keywords:** Climate sciences, Natural hazards, Solid Earth sciences

## Abstract

With global warming, the carbon pool in the degradation zone of permafrost around the Arctic will gradually be disturbed and may enter the atmosphere in the form of released methane gas, becoming an important factor of environmental change in permafrost areas. We selected the northwestern section of the Xiao Xing'an Mountains in China as the study area, located in the degradation zone on the southern margin of the permafrost region in Eurasia, and set up multiple study monitoring areas equipped with methane concentration sensors, air temperature sensors, pore water pressure sensors and soil temperature sensors for long-term monitoring of data changes using the high-density electrical method, ground penetrating radar and on-site drilling to survey the distribution of frozen soil and geological conditions in the study area, combined with remote sensing images of Sentinel-2 L1C and unmanned aerial vehicle photographs and three-dimensional image reconstruction, analysis of fire activities and related geological environmental factors. The results show that since 2004, the permafrost thickness of the marsh wetland in the study area has gradually reduced and the degradation rate obviously accelerated; the organic matter and methane hydrate (metastable methane hydrate and stable methane hydrate) stored in the permafrost under the marsh wetland are gradually entering the atmosphere in the form of methane gas. Methane emissions show seasonal changes, and the annual methane emissions can be divided into three main stages, including a high-concentration short-term emission stage (March to May), a higher-concentration long-term stable emission stage (June to August) and a higher-concentration short-term emission stage (September to November); there is a certain correlation between the change in atmospheric methane concentration and the change in atmospheric pressure and pore water pressure. From March to May every year (high-concentration short-term emission stage), with snow melting, the air humidity reaches an annual low value, and the surface methane concentration reaches an annual high value. The high concentration of methane gas entering the surface in this stage is expected to increase the risk of wildfire in the permafrost degradation area in two ways (increasing the regional air temperature and self-combustion), which may be an important factor that leads to a seasonal wildfire frequency difference in the permafrost zone of Northeast China and Southeast Siberia, with the peak in spring and autumn and the monthly maximum in spring. The increase in the frequency of wildfires is projected to further generate positive feedback on climate change by affecting soil microorganisms and soil structure. Southeastern Siberia and northeastern China, which are on the southern boundary of the permafrost region of Eurasia, need to be targeted to establish fire warning and management mechanisms to effectively reduce the risk of wildfires.

## Introduction

Methane gas released into the atmosphere in permafrost regions is generally believed to be derived from microbial gases released from melted sediments or local release of deep heat source gas^1–10^. Earth's cryosphere constitutes a vast climate-sensitive carbon reservoir that exists not only in the form of permafrost soil carbon but also in the form of a methane reservoir stratum under permafrost and ice sheets^11–14^. The estimated carbon storage of methane hydrate in the world is approximately 2 × 10^16^ m^3^, which is more than twice the total carbon of the world’s proven conventional fossil fuels. The energy density of methane hydrate is very high: 1 m^3^ of methane hydrate can decompose 164 m^3^ of methane gas under ideal conditions^15^. Drilling for permafrost in Siberia revealed that permafrost is not completely impermeable for gas but can hold gas accumulations. Deep methane hydrate stable zones exist in permafrost areas, and a considerable part of permafrost gas is concentrated in the form of metastable hydrates^4,15^. Climate change threatens the stability of this huge carbon pool; since 2007, global atmospheric methane concentrations have continued to rise after maintaining stability between 1990 and 2006^16,17^, and the area of the gas hydrate stability zone (GHSZ) is gradually shrinking in many regions^18–20^. The high latitudes in the Northern Hemisphere have experienced a more pronounced warming process than other regions. In the Arctic, North Atlantic, and North Pacific regions, methane gas is being released from unstable hydrates^21–25^.

Forest fires burn 50,000–200,000 hectares of boreal forest every year, and up to 80% of the boreal forests are located in the polar permafrost zone^26–28^. Approximately half of the world's soil carbon is located in permafrost in the Northern Hemisphere, but it is expected that a quarter of this permafrost will have thawed by 2100^29^. Melted permafrost will produce significant positive feedback on climate change by breaking down previously frozen organic matter and releasing greenhouse gas^30^. Wildfires in northern Eurasia mostly occur in summer because the temperature is higher at this time^31–33^, and the area of wildfires in central and eastern Siberia is the largest in June-July. However, wildfires in southeastern Siberia have peaks in spring and autumn; the area of wildfires in spring is the largest and the peak in spring is approximately four times the peak in autumn^32–34^. Study shows that forest fires in southeastern Siberia have increased in recent decades and are closely related to air temperature, drought index and incoming solar radiation^33^. There have been many fires in permafrost areas in Southeast Siberia and Northeast China (100°–150°E, 45°–55°N). With the southern boundary of permafrost gradually moving northward, wildfire may increase in permafrost degraded areas, and there is a lack of understanding about the influence of methane gas released from permafrost on temperature and the relationship between methane gas combustibility and wildfire.

To further study the rule of methane emission and its relationship with the frequency of wildfire in permafrost regions, we selected the northwestern section of the Xiao Xing'an Mountains in China as the study area, located in the degradation zone on the southern margin of the permafrost region in Eurasia. We set up multiple study areas equipped with methane concentration sensors, air temperature sensors, pore water pressure sensors and soil temperature sensors to track long-term monitoring data changes. We used the high-density electrical method (HDR), ground penetrating radar (GPR) and on-site drilling to survey the distribution of frozen soil and geological conditions in the study area, combined with remote sensing images of Sentinel-2 L1C and UAV photographs and three-dimensional image reconstruction, analysis of fire activities and related geological environmental factors.

## Results

### Permafrost distribution, degradation and methane hydrate sources in Northeast China

**Figure 1:**
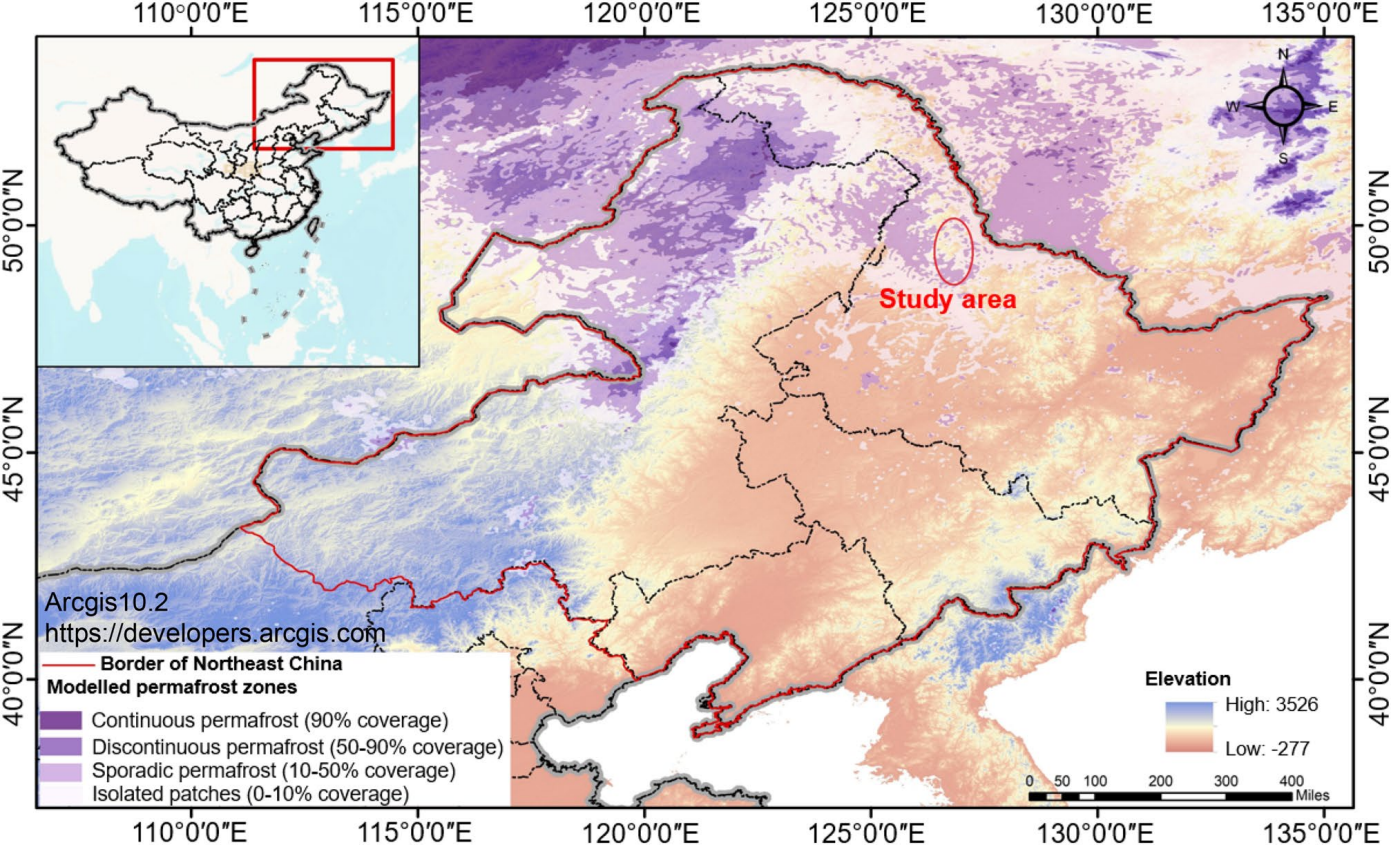
Distribution map of permafrost in Northeast China and location of the study area, according to the degree of permafrost coverage, divided into continuous permafrost (> 90%), discontinuous permafrost (50–90%), sporadic permafrost (10–50%), isolated patches (0–10%); data from Jaroslav O. et al.^36^, Arcgis10.2 https://developers.arcgis.com.

The permafrost area in southeast Siberia and Northeast China is located on the southern edge of the permafrost zone in Eurasia, in the transition zone between the cold temperate zone and the middle temperate zone, and is affected alternately by high and low pressure and monsoons of the inland region and the ocean. The annual average temperature is low, and the annual range is large, corresponding to a continental monsoon climate. Due to the control of the Siberian high pressure in winter, a stable inversion layer is widely distributed in this area, which has an important influence on the development and regional distribution of frozen soil. The permafrost in the area is distributed mainly in river valleys, foothills, shady slopes, etc., with typical “Xing'an-Baikal” permafrost distribution^35^. The thickness of frozen soil ranges from several meters to more than 100 m. Under the combined influence of regional geological conditions, climate change and human activities, the permafrost is degrading, with a transition of continuous permafrost to discontinuous permafrost to sporadic permafrost and even isolated patches from north to south^36^ (Fig. 1). The permafrost area in Northeast China is approximately 354,700 km^2^. The permafrost layer is characterized by a high temperature, low thickness and unstable thermal state, which is easily disturbed by the external environment, climate and human factors^37–42^. In addition, the mean annual air temperature (MAAT) growth in Northeast China is higher than that in the global MAAT^43–45^, which makes the region more susceptible to temperature changes. Since 1980, the MAAT fluctuation in Northeast China has increased, and the high-latitude region has experienced more extreme warming^46^. In particular, the northern Da Xing'an Mountains and Xiao Xing'an Mountains have experienced the most dramatic temperature increase^47^. Among them, the northern slope of the Da Xing'an Mountains is a more developed area of permafrost in northeastern China. The permafrost in this area has degraded, and the melting zone has expanded significantly^48^. Through the observation points of permafrost along the Ituri River, it is found that the mean annual ground temperature (MAGT) of permafrost has been increasing due to the influence of the surrounding temperature rise. In 1984–1997 and 1997–2010 in particular, the depth of 13 m increased by 0.2 °C and 0.4 °C, respectively^49,50^. The ground temperature at a depth of 10 m in Mohe County in northern Heilongjiang Province rose from − 3.7 °C in 1975 to − 1.5 °C in 1978^51–53^. In the past 50 years, the temperature in Northeast China has generally increased by 0.9–2.2 °C, which has caused the southern edge of the permafrost to move northward. The area of permafrost has decreased by 35–37% compared with that in the 1970s. If the temperature increases by 1–1.5 °C over the next 40–50 years, the southern edge of permafrost will move further northward, and the area of permafrost may decrease by 35%^42^.

Siberia and Northeast China's permafrost regions have formed large-scale sedimentary basins during multiple geological changes, and sedimentary layers of different depths have been formed in different geological periods, including marine sedimentary layers and continental sedimentary layers formed by ancient ocean movements. The Late Devonian Xing'an trough (between the Xiao Xing'an Mountains and Jiamusi) is uplifted, the plates are gradually amalgamated, the sea water gradually deepens from west to east, and the sedimentation transitions from continental to marine. At the end of the Late Permian, the paleo Asian Ocean basin was completely closed, forming a continental sedimentary environment and forming a vastly thick layer of continental clastic rocks and volcanic sedimentary combinations. The Carboniferous-Permian has the most extensive marine and continental sedimentary layer in Northeast China^54,55^. The marine sedimentary basins have relatively stable tectonic activities, fewer types of sedimentary facies than other regions, fewer changes in oil-generating rocks and oil-storing rocks, rich oil and gas resources, and timely migration to high-quality reservoirs, conducive to the formation of large-scale structural oil and gas reservoirs^56^. However, most of the continental sedimentary basins are located in the piedmont and intermountain active areas, which are relatively small in scale. These areas are often affected by orogenic activities and faulting activities, and the oil reservoir preservation conditions are relatively unstable. However, regardless of whether they are aquatic or terrestrial, the organic matter of animals and plants can be used as the source material of oil, and their quantity and organic matter undergo the conditions for conversion into hydrocarbons, and the continental sedimentary environment is no less productive than the marine sedimentary environment, potentially generating oil and gas fields. The Daqing Oilfield is a representative continental sedimentary oil field in Northeast China^57–60^. Large-area sedimentary basins provide sufficient material conditions for the formation of these methane hydrates in this region.

Hydrate deposits associated with permafrost are usually found in high-permeability rock formations in areas of known petroleum systems, which provide a certain depth of gas source and can sometimes be displaced laterally from the hydrate deposits, while the permafrost layer formed in the multiple ice ages hinders the movement and leakage of gas and seals it in the permafrost, and the formation of permafrost layer also provides the conditions for the formation of methane hydrate at low temperature and high pressure and blocks the interference of the external environment on the storage of methane hydrate^61,62^.

There are montmorillonite layers in the permafrost area of Northeast China, and montmorillonite is considered one of the traces of natural gas hydrate, with good water swelling, adhesion and adsorption properties^63,64^. The results show that methane molecules can enter montmorillonite and form stable hydrates. Compared with pure water systems (295.7 kPa and 8.28 mPa), natural gas hydrate with montmorillonite has a higher temperature and lower steady pressure (296.1 kPa and 5.54 mPa). The presence of montmorillonite further promotes the formation of methane hydrate^65,66^.

A large amount of methane hydrate reserves have been found under permafrost in the Mohe Basin, which is located in northern Northeast China^67^, and the range of methane hydrate reserves in the stable zone is approximately 0.49 × 10^12^–0.79 × 10^12^ m^3^. In permafrost regions, in addition to the deep stable methane hydrate, shallow metastable methane hydrates exist in ice-containing permafrost sediments because the ice layer blocks methane hydrate breakdown^12,68,69^. With the degradation of permafrost areas, these residual methane hydrates will be released earlier.

### Climate change in the study area and temperature change of the permafrost at monitoring points

The study area is located in the permafrost swamp area of section k153–k183 of the Bei'an-Heihe expressway, located in the Xiao Xing'an Mountains on the edge of the Sunwu-Jiayin Basin (integrated with the Breya-Gaya Basin in Russia to the north), China. The location is between 127°17′31″E, 127°21′24″E and 49°30′57″N, 49°41′50″N (Fig. 1). This area belongs to low mountain and hilly landform area, with an altitude between 110 and 755 m. There are cold temperate coniferous and broad-leaved mixed forests at high altitudes, moss grass grows on the lower surface, and thick peat soils are distributed on the soil surface. Swamp wetlands and permafrost are distributed mostly in relatively low-lying areas and floodplains, with thicknesses of approximately 5–10 m, average ground temperatures of − 0.5 ~  − 5 °C, and melting permafrost areas of more than 80%. Our study team found that there was abnormal growth of herbaceous plants and seasonal occurrence of wildfires, which was evaluated to be caused by abnormal greenhouse gas emissions.

**Figure 2:**
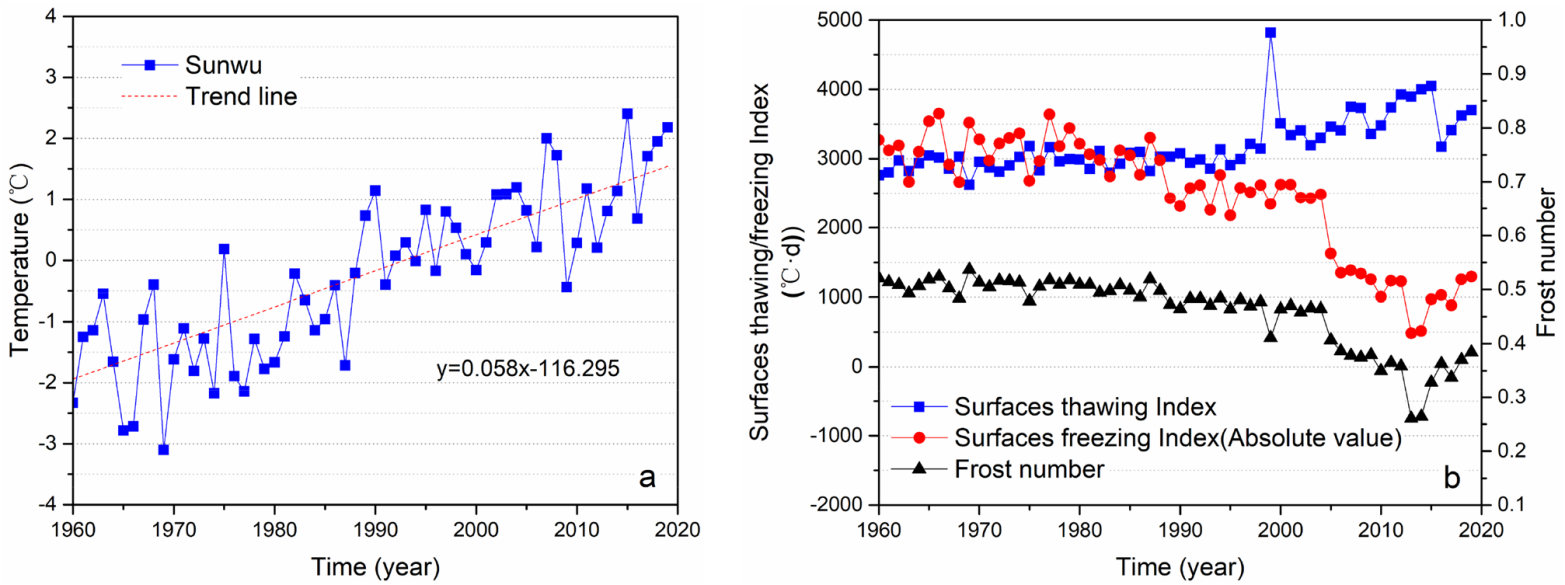
Changes in air temperature, surface freezing (thawing) index and surface frost number in the study area from 1960 to 2019. (**a**) Air temperature change curve in the study area, (**b**) Surface freezing, thawing index and frost number change curve; the data come from Sunwu National Meteorological Station near the study area (http://data.cma.cn).

According to the air temperature change curve of Sunwu National Meteorological Station near the study area from 1960 to 2019, it can be seen that the overall air temperature in the study area is increasing; its warming rate is 0.58 °C/10 a (Fig. 2a), and the average temperature rose above 0 °C in 1999. In addition, through the surface freezing index, surface frost number and surface thawing index change curves near the study area (Fig. 2b), it can be seen that the surface thawing index has increased significantly since 2004, and the surface freezing index and surface frost number have shown a significant downward trend. There was a cliff-like decline during 2004–2006, and it is inferred that the remaining shallow permafrost in the study area may have completely thawed^70^.

**Figure 3:**
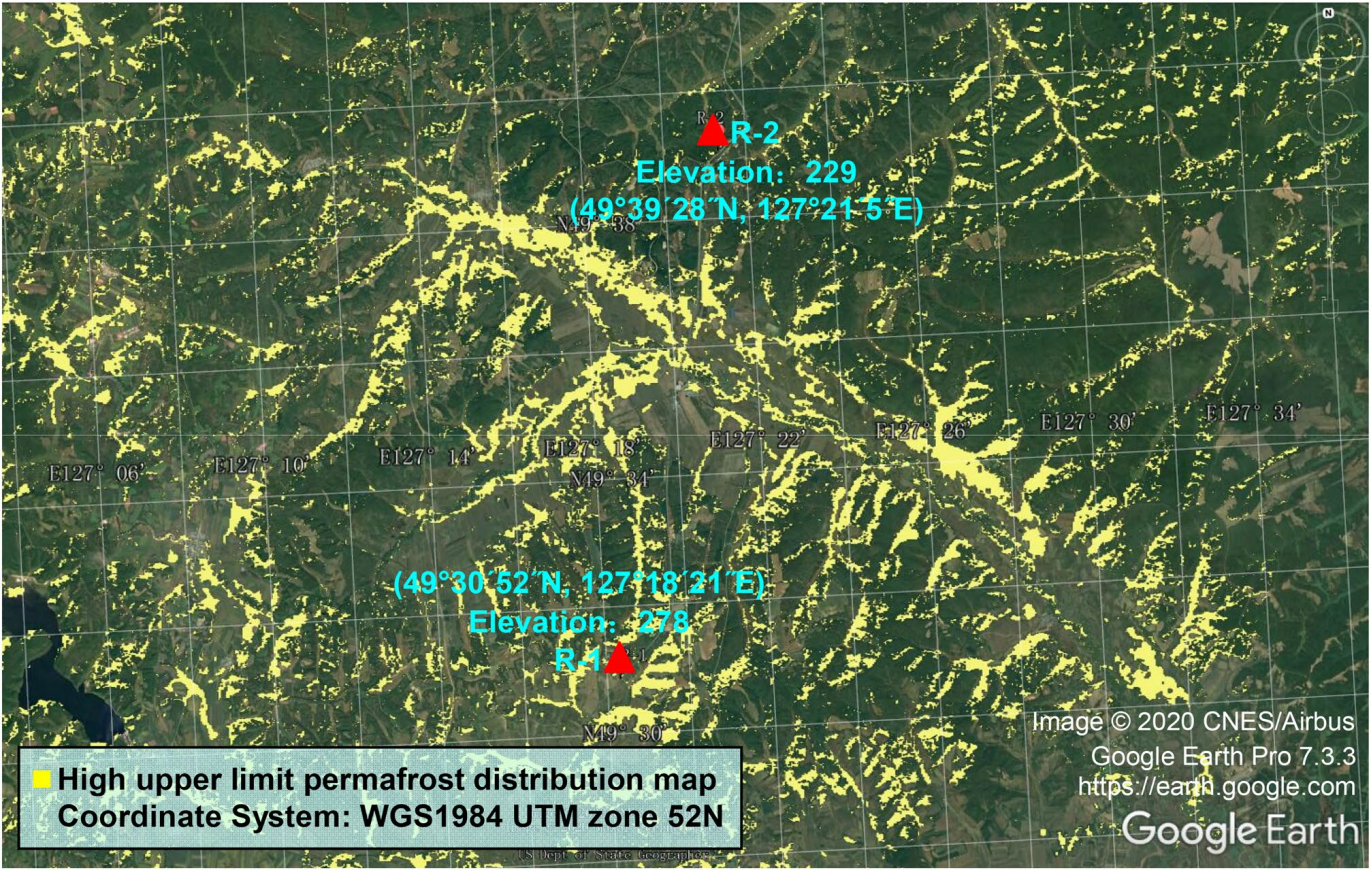
Location coordinates of study area R-1 and study area R-2 and distribution of frozen soil nearby; data from Wang et al^35^, Landsat7 single-band image data processed by ArcGIS and imported into Google Earth Pro 7.3.3 https://earth.google.com.

**Figure 4:**
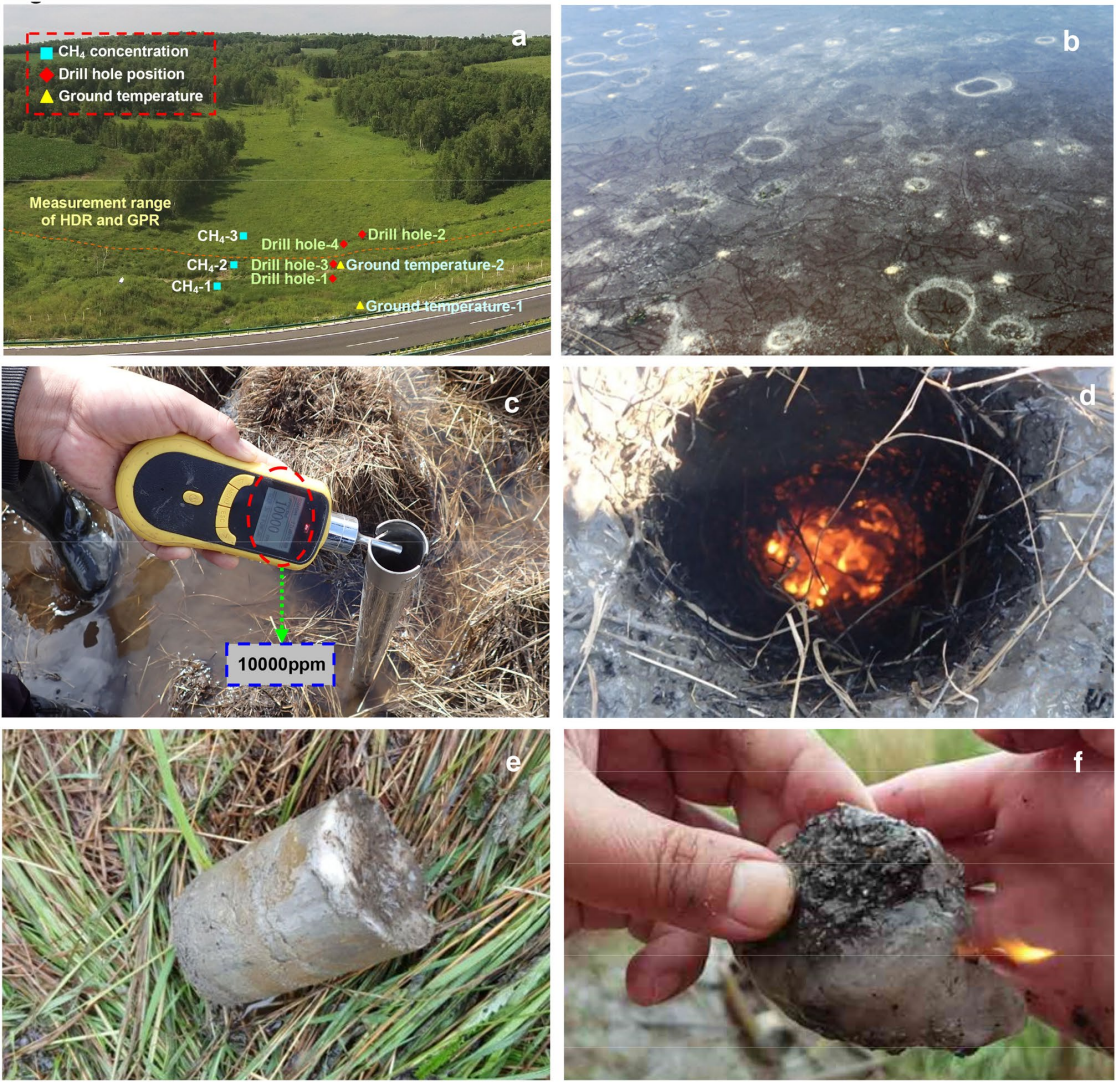
Drilling results and combustible ice samples in study area R-1. (**a**) Location of the drill hole, the surface methane concentration sensor, the ground temperature sensor, and the measurement range of the high-density electrical method (HDR) and the ground-penetrating radar (GPR) in the study area R-1, (**b**) High-concentration methane bubbles under the ice surface at the drilling site in spring, (**c**) High-concentration methane gas discharged along the drill pipe as found by a light drilling machine near borehole 4 (up to 10,000 ppm measuring instrument upper limit; the measuring instrument is an SKZ1050-CH4 methane detector), (**d**) The flame formed in contact with a fire source after a hole was drilled at a depth of 15 m, (**e**) Combustible ice samples obtained by drilling 8 m underground at drill holes-3, (**f**) The burning process of Combustible ice samples on site.

We chose the permafrost swamp zone on the left side of the K161 + 300–K161 + 900 and K177 + 400–K177 + 800 road sections of the Bei'an-Heihe expressway, set up the study monitoring areas R-1 (49°30′52″N, 127°18′21″E, altitude 278 m) and R-2 (49°39′28″N, 127°21′5″E, altitude 229 m), respectively (Fig. 3), and configured a methane concentration sensor, air temperature sensor, soil pore water pressure sensor and ground temperature sensor in R-1. Since June 2015, we have monitored the changes in related parameters in R-1 and adopted high-density electrical method (HDR) and ground penetrating radar (GPR) exploration and multiple on-site drilling efforts (Fig. 4a) to obtain combustible ice samples (Fig. 4e,f) in the permafrost layer 8 m from the surface. After the drill bit penetrated the first layer of the underwater ice layer (1.5–2 m from the surface), the high-concentration methane gas was rapidly discharged, the surface methane concentration reached the upper limit of instrument measurement (10,000 ppm) (Fig. 4c), and the drill hole formed a flame of up to 1 m after contacting the fire source (Fig. 4d). In addition, a large number of bubbles were found under the ice surface in the water accumulation area near the drilling position, and the bubbles contained high concentrations of methane gas (Fig. 4b).

**Figure 5:**
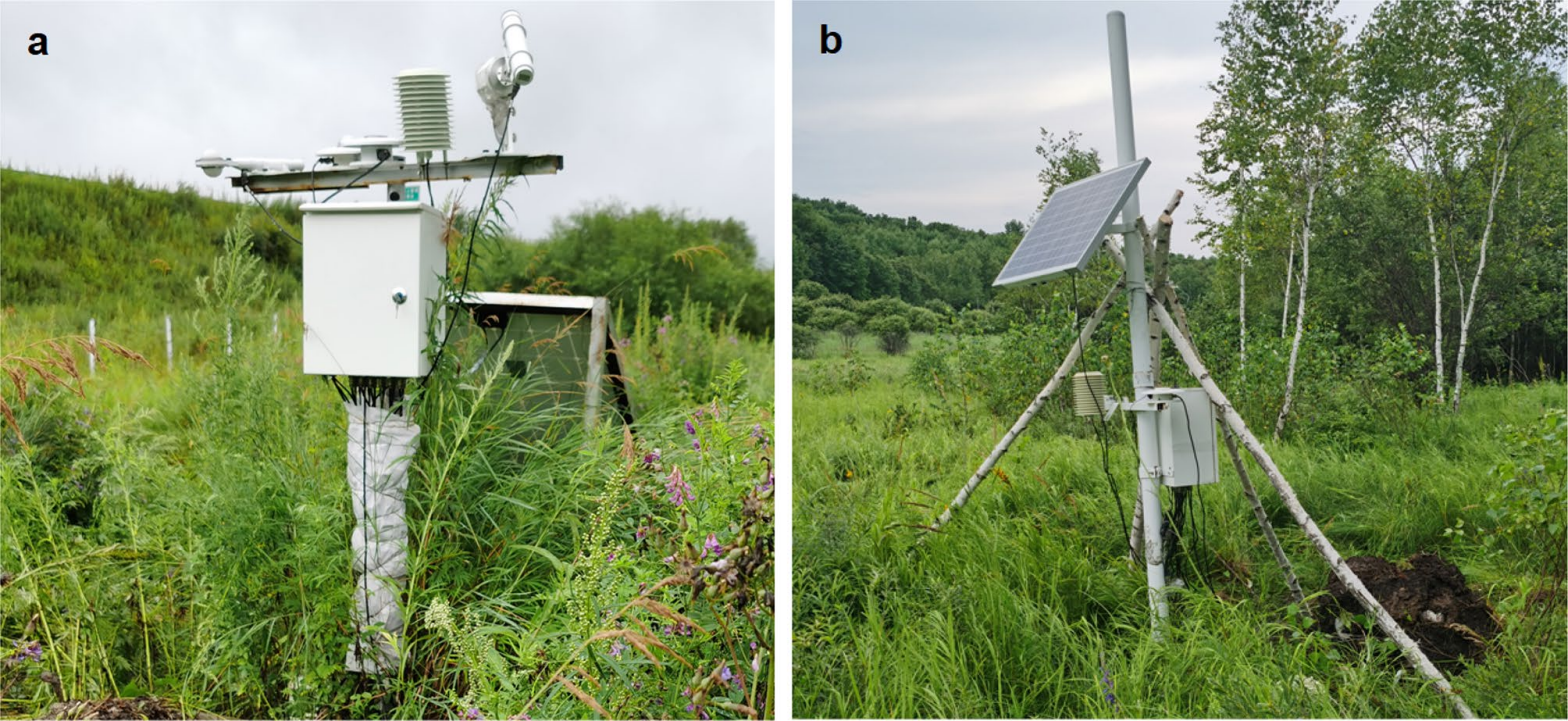
R-1 and R-2 meteorological stations in the study area. (**a**) Meteorological station in the study area R-1, (**b**) Meteorological station in the study area R-2. Meteorological station is composed mainly of a data acquisition host, data analysis system, data transmission system, Internet of Things sensors, GPRS wireless transmission system, solar power supply system, etc.

This paper adopts the meteorological monitoring system (http://www.qixiangshuju.com) and the ground soil physical parameter monitoring system (http://www.hzjly.cn) to monitor the meteorological data in real time on site. The meteorological station model is GD24-YCXQ, which mainly includes surface meteorological element sensors such as surface methane concentration sensors (QT21-BX80-CH_4_) and air temperature sensors (GD51-KWSY). The soil physical parameter monitoring system uses a soil pore water pressure sensor (KXR-3034) and a soil temperature sensor (HJ6-FM-TWB) to monitor related parameters. The meteorological stations in the study area R-1 and R-2 are shown in Fig. 5. The above data monitoring systems all adopt the GPRS communication mode, which transmits monitoring data to the central station regularly and can automatically generate messages without manual intervention, with high precision and high reliability. In addition, we monitor the operation status of the meteorological station and ground soil physical parameter monitoring system in the study area based on the data upload status.

Through sampling soil samples of different depths of four drill holes in study area R-1, it is found that the main components of the soil layer in the range of drill hole depth from top to bottom are as follows. (1) Peat soil, which contains incomplete decomposed grass roots and is a highly compressible and frost heaving soil, with the frozen part presenting a layered and patchy frozen soil structure. (2) Silty clay, which is a highly compressible and frost heaving soil, with the frozen part presenting a layered and patchy frozen soil structure. (3) Gravel sand, where the mineral composition is mainly volcanic rock debris, mixed quartz feldspar montmorillonite, etc., the pores are filled with clay, and the frozen part is a microlayered and layered frozen soil structure. (4) Fully weathered mudstone, where the mineral composition is mainly volcanic debris and mixed quartz feldspar, the pores are mainly filled with clay, containing clay interlayers, and the frozen part is a microlayered and layered frozen soil structure (Table 1).

**Table 1:** Soil samples in different depths at each drilling position.

No./depth	Soil samples	Soil sample composition	No./depth	Soil samples	Soil sample composition	No./depth	Soil samples	Soil sample composition	No./depth	Soil samples	Soil sample composition
No.1 4.0 m		0–4 m Peat soil	No.2 1.3 m		1.3 m Peat soil	No.3 1.1 m		0.4 m Grass root	No.4 0.5 m		0.5 m Peat soil
No.1 5.0 m		5 m Sandstone and small mudstone	No.2 3.5 m		3.5 m Peat soil	No.3 2.7 m		1.3 m Peat soil	No.4 2.0 m		2 m Peat soil and sand
No.1 6.0 m		6 m Coarse sand and slightly mudstone	No.2 4.5 m		4.5 m Sandstone and fine sand	No.3 4.6 m		2.7 m Peat soil and gravel	No.4 3.0 m		3 m Peat soil
No.1 7.0 m		7 m Fine and small crushed mudstone	No.2 6.5 m		6.5 m and 7.4 m Sandstone	No.3 4.6 m		4.6 m Sandstone	No.4 4.0 m		4 m Peat soil
No.1 8.0 m		8 m Coarse and small crushed mudstone	No.2 7.4 m		8.5 m Sandstone and fine sand	No.3 7.0 m		7 m Sandstone and small mudstone	No.4 5.0 m		5 m Sandstone
No.1 9.0 m		9 m Massive mudstone	No.1 8.5 m		9 m Massive mudstone	No.3 9.0 m		9 m Massive mudstone	No.4 7.0 m		7 m Sandstone

Soil samples composition with different depths in research area R-1, the drilling position is shown in Fig. 4a.

**Figure 6:**
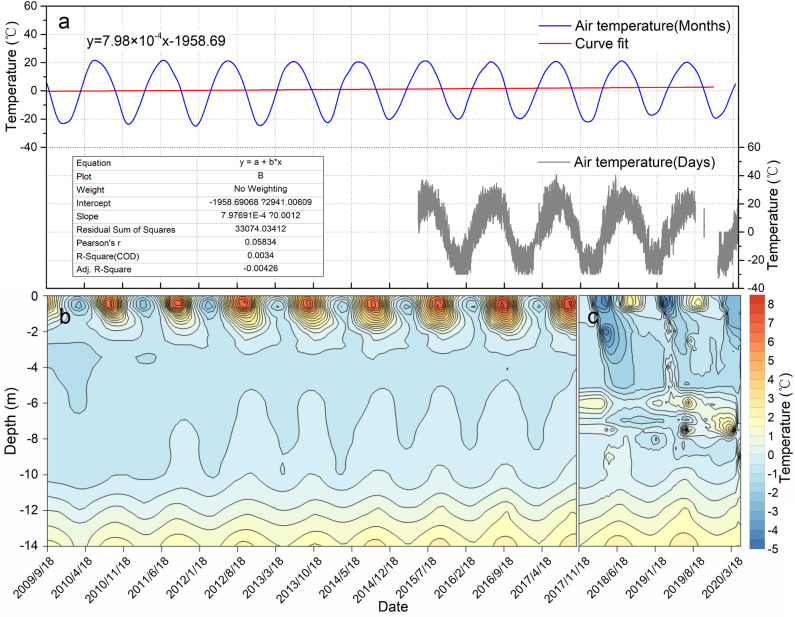
Changes in air temperature and ground temperature in study area R-1. (**a**) Change curve of the monthly average value and the real-time value of the air temperature. (**b**) Contour map of the 0–14 m deep ground temperature at monitoring point 1. Since monitoring point 1 is close to the expressway, the surface temperature is very strongly affected by the roadbed. (**c**) Contour map of the 0–14 m deep ground temperature at monitoring point 2.

Changes in ground temperature caused by climate warming and engineering activities are important disturbance factors for the degradation of permafrost. The monitoring results of air temperature and ground temperature in the study area show that the overall air temperature is increasing, and as the air temperature changes, the ground temperature peaks around September each year, and the peak gradually decreases with increasing depth. From September 18, 2009 to the present, the ground temperature at different depths at monitoring point 1 in study area R-1 has shown a gradual upward trend, and the upper and lower limits of permafrost are gradually changing with increasing ground temperature. The thickness of the frozen soil layer is gradually decreasing. Among the monitoring points, because point 1 is close to the expressway, the ground temperature is relatively high due to the influence of the roadbed. When the ground temperature monitoring started on September 18, 2009, the upper limit depth of frozen soil was 1.55 m, the lower limit depth was 11.25 m, the thickness of frozen soil was 9.7 m, and the minimum temperature was − 1.2 °C. On September 25, 2016, the upper limit depth of frozen soil was 2.05 m, the lower limit depth was 8.75 m, the frozen soil thickness was 6.7 m, and the minimum temperature was − 0.6 °C. From the overall change trend of ground temperature at monitoring point 1 in study area R-1, it can be seen that the permafrost layer shows a severe degradation trend under the influence of climate change and engineering construction, which is manifested by a significant falling in the upper limit, a significant rising in the lower limit, degradation mode of the frozen soil is the simultaneous change in upper and lower limits, and a lower limit rising speed higher than the upper limit falling speed^70^. The permafrost layers in the study area are all high-temperature frozen soils, which may further degrade and disappear completely in the next few years (Fig. 6). The monitoring results are consistent with the analysis results of Fig. 2b. Since October 15, 2017, we added ground temperature monitoring point 2 to study area R-1 and found that ground temperature is also gradually increasing, and a complex positive temperature layer has appeared in the depth range of 6–8 m from the surface. This may be caused by the rapid accumulation of methane gas decomposed by methane hydrate in permafrost in the soil pores at different locations; the pressure increase caused by gas accumulation and compression would cause the soil temperature to rise.

### Methane emission law and mechanism in the study area

**Figure 7:**
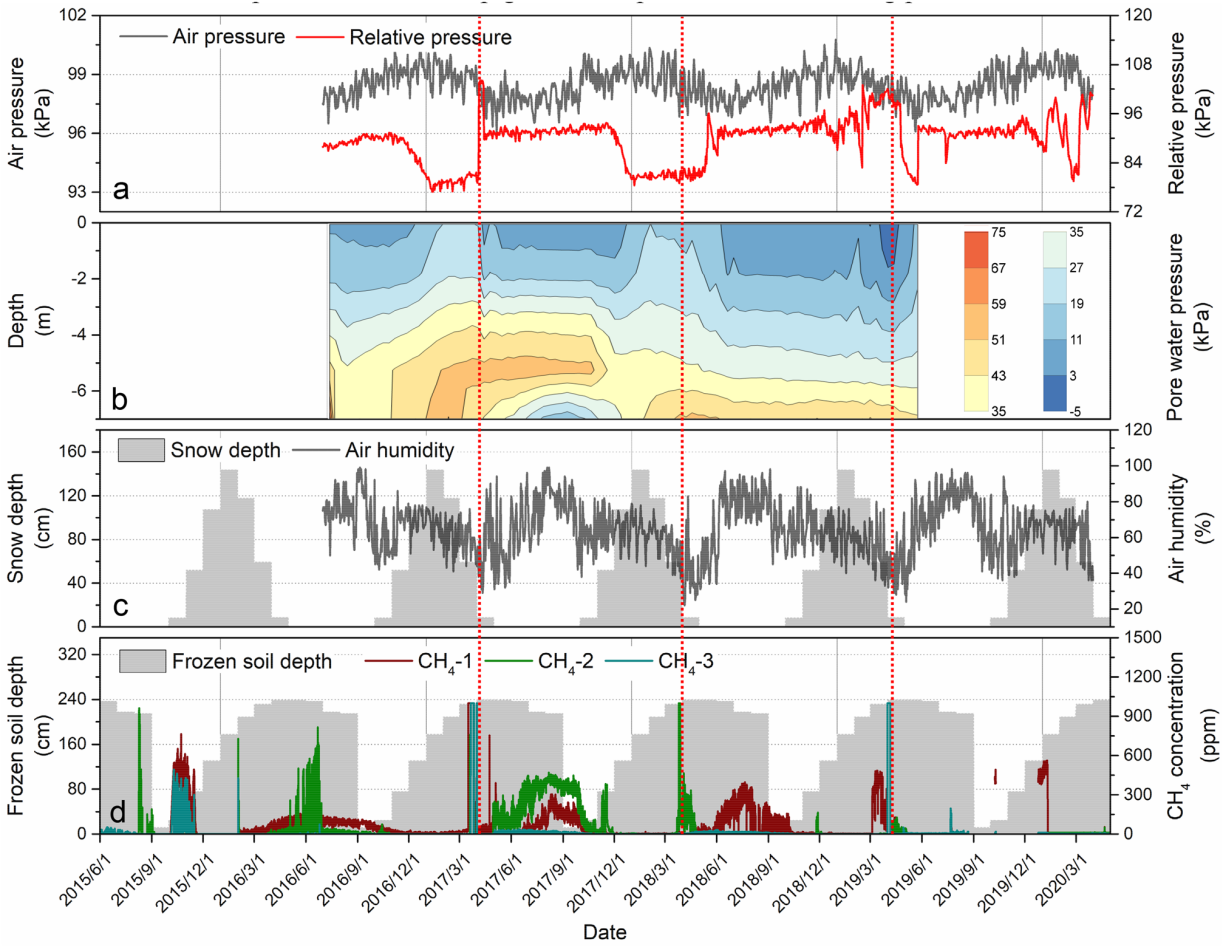
Changes in monitoring factors in study area R-1. (**a**) Daily value of atmospheric pressure and the relative pressure change curve at a depth of 1 m underground in drill hole-4, (**b**) Contour map of pore water pressure at a depth of 0–7 m in drill hole-4, (**c**) Changes in atmospheric relative humidity and average snow thickness, (**d**) Changes in surface methane concentration and the monthly maximum active layer thickness (frozen soil depth, distance from the surface to the upper limit of frozen soil) at three monitoring points. The red dashed line is the auxiliary line for the maximum methane concentration in spring.

The monitoring of R-1 in the study area shows that the atmospheric pressure *P*_a_ changes periodically with the seasonal variation, and the atmospheric pressure *P*_a_ reaches the maximum value in winter every year, decreases gradually in spring and reaches the minimum value in summer (Fig. 7a). However, due to gas accumulation and ice-water phase transition, the soil pore water pressure *P*_w_ gradually increased in winter and reached its maximum in spring (Fig. 7b), and from 2017 to 2019, the increasing range of pore water pressure at different depths generally decreased year by year, which may be caused by the gradual thawing of permafrost. The results of the three monitoring points for surface methane concentration show that the annual maximum value of surface methane concentration occurs in spring every year (up to the upper limit of 1000 ppm of the sensor). In 2017, all three monitoring points had annual maximum values in spring, while in 2018, only monitoring point 2 had annual maximum values in spring, and in 2019, only monitoring point 3 had annual maximum values in spring. The overall surface methane concentration decreases year by year (Fig. 7d), which may be caused by the gradual decrease in soil carbon after the thawing of frozen soil. In addition, the pressure gradient is an important driving force in the process of methane gas migration, which directly determines the speed of methane gas entering the atmosphere through the soil layer. When methane enters the atmosphere, the variation in pore water pressure and atmospheric pressure determines the speed of methane entering the atmosphere. We conducted statistical analysis on the surface methane concentration and atmospheric pressure *P*_a_ and found (Fig. 9a) that the surface methane concentration is negatively correlated with the atmospheric pressure as a whole, and the maximum occurrence of methane concentration in spring is near the maximum pore water pressure *P*_w_ (Fig. 7a); that is, when the atmospheric pressure is low and the pore water pressure is high, methane gas may enter the atmosphere more easily, so we introduce the relative pressure *P*_r_ to describe the main release power of methane gas into the atmosphere Eq. (1). We conducted statistical analysis on the surface methane concentration and relative pressure *P*_r_ and found (Fig. 9b) that the smaller relative pressure *P*_r_ will promote the release of methane gas, and the methane gas release stage is mainly concentrated in three ranges (the first range is 79–83 kPa, the second range is 89–94 kPa, and the third range is 97–104 kPa. The second range (89–94 kPa) has the largest methane gas release, which is probably due to the increase in temperature during this period, and the gradual thawing of snow on the surface accelerated gas release. When the relative pressure is lower than 89 kPa, there is a frozen layer on the surface at lower temperature, and it is difficult for methane gas to enter the surface. (Fig. 7c).

1 $$ P_{{\text{r}}} = P_{{\text{a}}} - P_{{\text{w}}} $$

where *P*_r_ is the relative pressure, *P*_a_ is the atmospheric pressure, and *P*_w_ is the pore water pressure at a depth of 1 m.

It can be seen from the surface methane concentration curve in the whole year that (Fig. 7d) the annual release of methane gas is mainly divided into three stages: the first stage of methane gas release is the high concentration short-term emission stage (March to May), and the peak value is approximately March 25 every year. Due to the ground snow and frozen layer block in winter (Fig. 7c), the methane gas in the soil is difficult to release and gradually accumulates under the frozen layer, and the sources of this part of methane gas mainly include methane hydrate and methanogens sealed in permafrost. When the temperature gradually rises in spring, the snow and frozen layers on the ground gradually thaw, and the smaller relative pressure *P*_r_ will promote the release of methane gas. The second stage of methane gas release is the higher concentration long-term stable emission stage (June to August). In this stage, the air temperature gradually rises to the highest level in the whole year, the action of methanogens in the swamp and the decomposition of methane hydrates in the frozen layer produces methane and superimposed emissions, and the surface methane concentration is high and continues to be stable. In addition, due to the higher temperature at this stage and the methanogenic bacteria being relatively active, the proportion of methane gas from methanogenic bacteria increased at this stage, but due to the relatively slow methanogenesis, the surface methane concentration at this stage was lower than that at the first stage. The third stage of methane gas release is the higher-concentration short-term emission stage (September to November). In this stage, the air temperature gradually decreased, the capacity of the swamp to produce methane decreased, the depth of the permafrost active layer reached its maximum value, the accumulated methane gas between the active layer and the upper limit of permafrost and the methane gas produced by the action of methanogens were gradually released, and the surface reappeared for a short time of higher methane concentrations.

**Figure 8:**
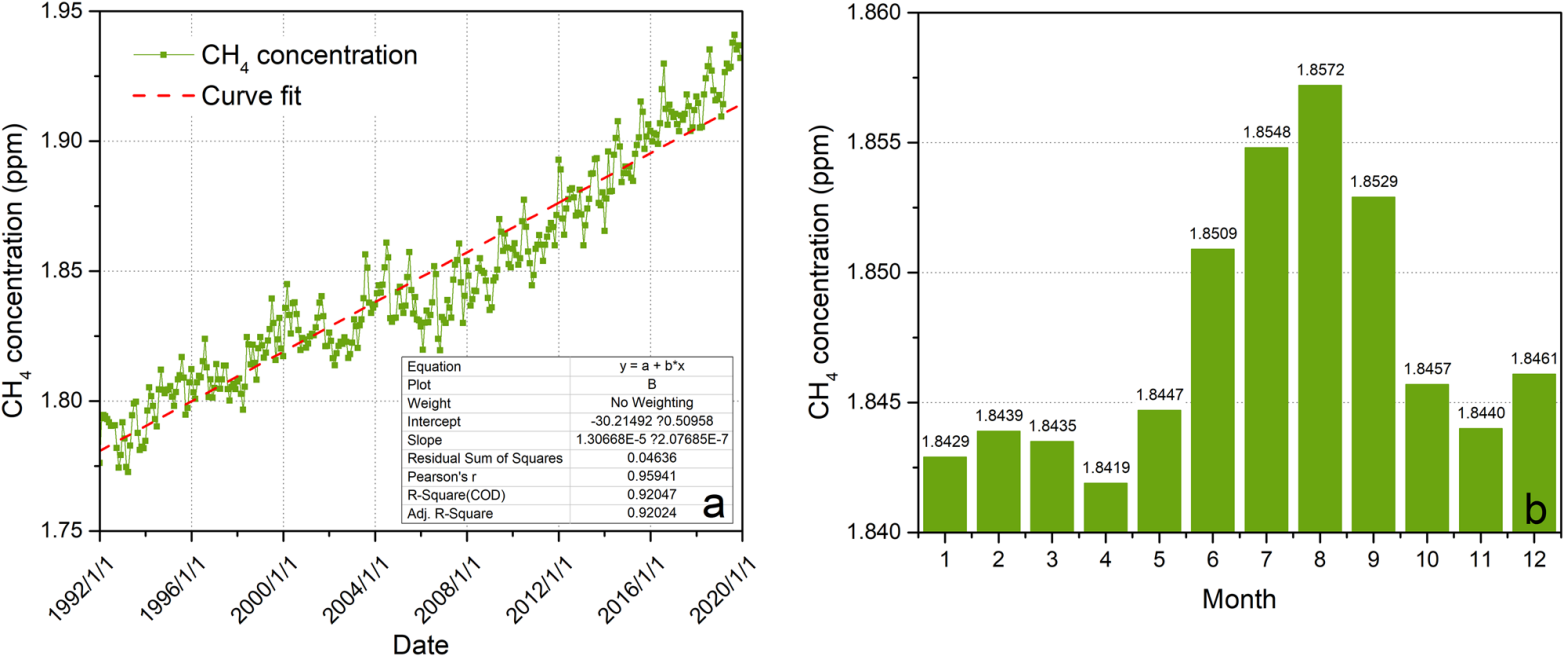
Air methane concentration in Waliguan. (**a**) Variation curve of atmospheric methane concentration in Waliguan from 1992 to 2019, (**b**) Monthly mean map of atmospheric methane concentration in Waliguan from 1992 to 2019.

**Figure 9:**
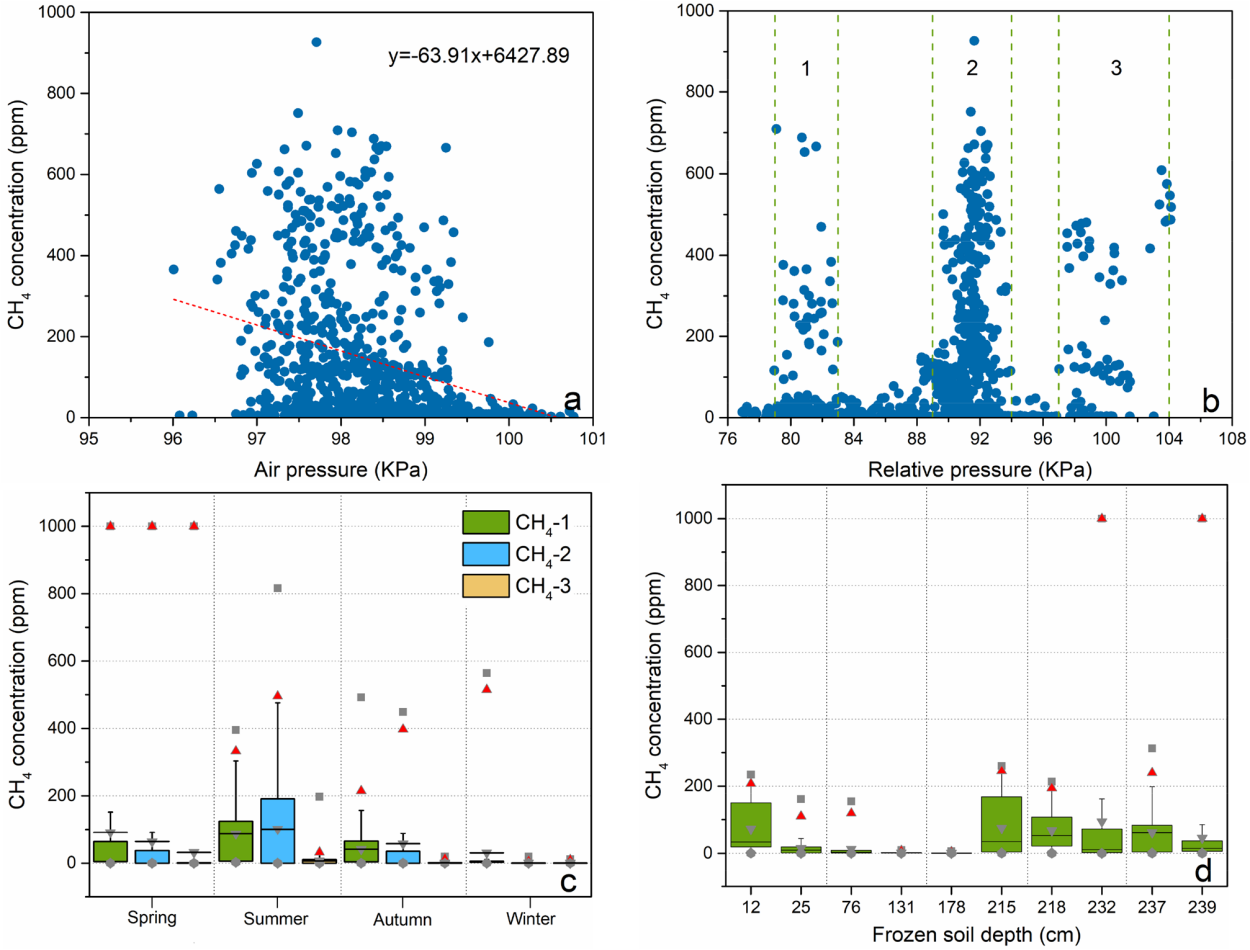
Methane gas release power in the study area. (**a**) The average daily methane concentration on the surface as a function of atmospheric pressure, where the dashed line is a linear regression fit from the observed data, (**b**) The average daily methane concentration on the surface as a function of relative pressure, where the green dashed line represents the decomposition auxiliary line for different distribution ranges, (**c**) Statistical analysis of the methane concentration at different surface monitoring points in different seasons, where the box diagram represents the 25th and 75th percentiles, the red triangle represents the 99th percentile, and the gray square represents the maximum value, (**d**) Statistical analysis of the methane concentration at different surface monitoring points in different monthly maximum active layer thicknesses (frozen soil depth, distance from the surface to the upper limit of frozen soil); the box diagram represents the 25th and 75th percentiles, the red triangle represents the 99th percentile, and the gray square represents the maximum value.

To compare the characteristics of methane emissions from permafrost regions in Northeast China and Waliguan, this paper uses the observation data of atmospheric methane concentrations from 1992 to 2019 of the Waliguan Global Meteorological Station for analysis. The Waliguan mountain belongs to the Qinghai Nanshan mountain system on the northeastern edge of the Qinghai-Tibet Plateau, with a northwest-southeast direction and a relative elevation difference of approximately 600 m. According to the observation curve of atmospheric methane concentration in Waliguan from 1992 to 2019 (Fig. 8a), the atmospheric methane concentration in Waliguan showed an upward trend from 1992 to 2002 and was basically stable from 2002 to 2006, and the overall period from 2007 to 2019 was one of slow growth, with the largest value in 2019, at an annual average of 1.9283 ppm. In addition, through the monthly average value of atmospheric methane concentration in Waliguan (Fig. 8b), it can be seen that the annual atmospheric methane concentration in Waliguan has a significant bimodal seasonal change feature, and the first peak occurs in summer (June to August), the maximum occurs in August at 1.8572 ppm, the second peak occurs in winter (December to February), and the maximum occurs in December at 1.8461 ppm. Based on the analysis of methane concentration in different seasons and permafrost depths of study area R-1, it is found that the maximum total emission of methane gas is in summer, which is consistent with the observation results in Waliguan. However, the maximum surface methane concentration of R-1 in the study area occurs in spring, while the total amount and concentration of methane emissions in autumn and winter are relatively small (Fig. 9c).

**Figure 10:**
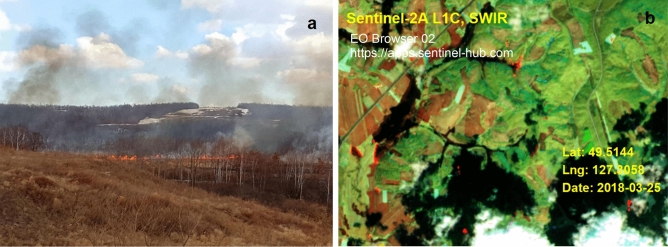
Burning situation of wildfires near the study area R-1. (**a**) Wildfire burning situation in the study area R-1 on March 22, 2017, (**b**) Wildfire occurring near the study area R-1 on March 25, 2018, obtained by Sentinel 2, where the red area is the burning area, EO-Browser 02 satellite image https://apps.sentinel-hub.com.

In addition, the atmospheric methane concentration in Waliguan showed an overall upward trend, while the maximum surface methane concentration in study area R-1 in spring decreased year by year, which may be due to the gradual reduction in soil carbon after thawing of frozen soil. In the stage of maximum methane concentration in spring, the air humidity is the lowest (Fig. 7c), a large number of wildfires appear near the study area in this stage (Fig. 10), and methane emission may be an important factor to promote wildfires. In addition, when the active layer thickness is large, the surface methane concentration is the highest, and when the monthly maximum active layer thickness increases to 232 cm and 239 cm, the maximum methane concentration reaches the measurement upper limit of 1000 ppm (Fig. 9d); it can thus be seen that the active layer thickness is an important factor promoting methane emission.

### Effect of methane gas emission on air temperature

**Figure 11:**
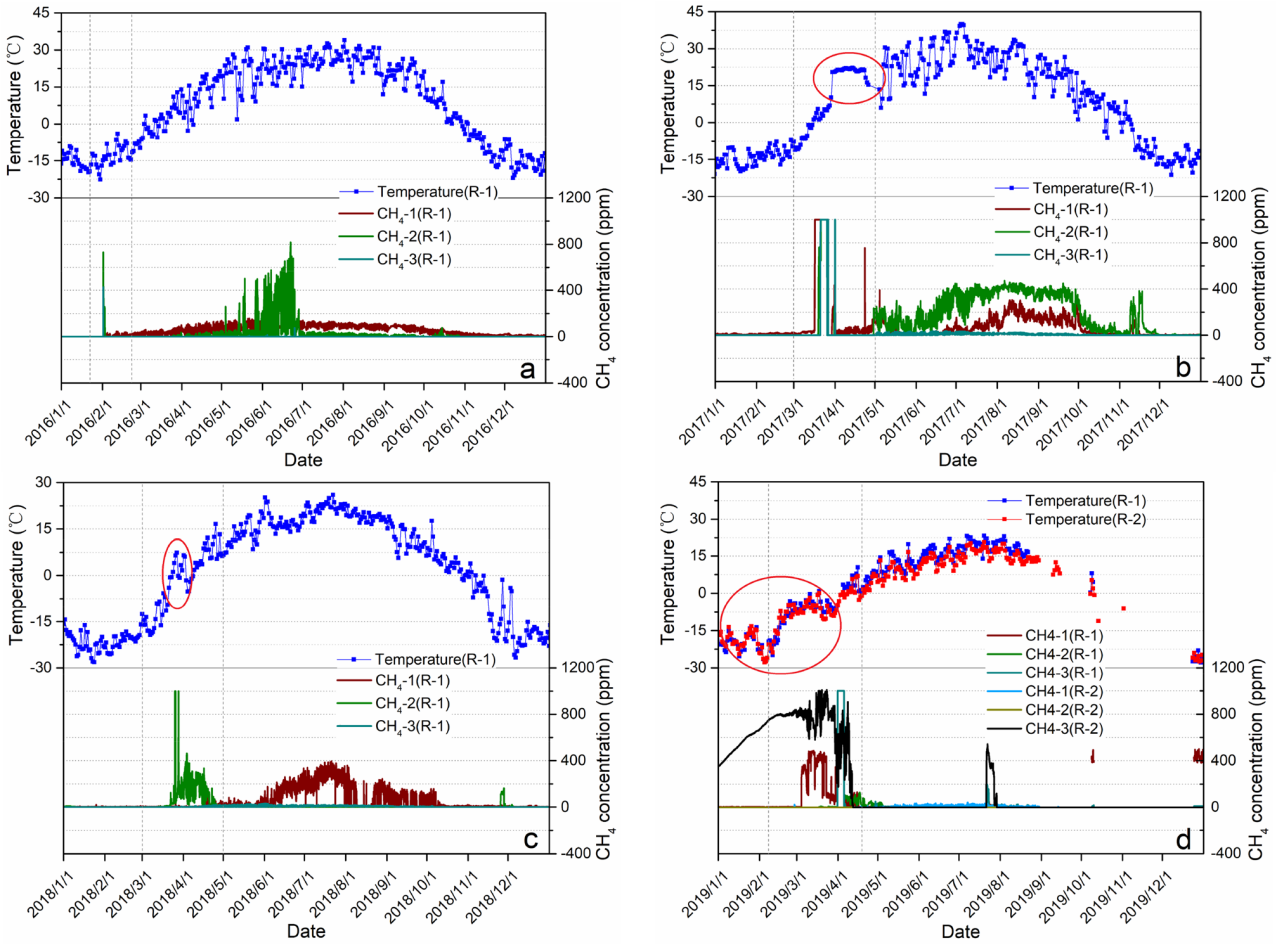
Daily mean curves of surface methane concentration and air temperature at study areas R-1 and R-2. (**a**) Daily mean curve of surface methane concentration and air temperature at R-1 in 2016, (**b**) Daily mean curve of surface methane concentration and air temperature at R-1 in 2017, (**c**) Daily mean curve of surface methane concentration and air temperature at R-1 in 2018, (**d**) Daily mean curve of surface methane concentration and air temperature at R-1 and R-2 in 2019.

To further study the impact of methane emissions on air temperature, we added a surface methane concentration sensor and air temperature sensor in the study area R-2 and began to monitor the surface methane concentration and air temperature of this study area in real time starting in January 2019. Combined with the monitoring results of R-1 and R-2 in the study area, it is found that (Fig. 11) in spring, when the surface methane concentration increases rapidly, there will be a peak phenomenon of rapid increase in the near surface air temperature, and the temperature change amplitude and change time are consistent with the methane emission time. Methane gas has a significant promotion effect on the increase in near-surface air temperature. However, due to regional differences, the study area R-2 also had high methane gas emissions from January to February.

Comparative analysis of air temperature from 2016 to 2018 shows that at a time when methane gas emissions are high, the amplitude of air temperature changes is greater. Due to the influence of wildfires, the air temperature peak in the spring of 2017 and 2018 increased more rapidly (Fig. 11b,c). In addition, the study area is located in the basin and valley area. There is a significant temperature inversion phenomenon, and the air moisture content is higher due to the presence of a certain amount of water in R-1, so the overall air temperature of R-1 is higher than that of R-2. However, comparing the air temperature between R-1 and R-2 in 2019, since the surface methane gas concentration of R-2 was higher than that of R-1 from January to May, the air temperature of R-2 at this stage is close to or even exceeds that of R-1 under the influence of methane gas (Fig. 11d).

### Effect of methane gas emissions on the frequency of wildfires

In recent years, global wildfires have become more frequent. In addition to Australia, West Africa, Amazon rainforest and other places, large-scale wildfires have also occurred in permafrost areas such as the Arctic and Siberia. Study shows that the increase in wildfires in permafrost areas is attributed to the increase in air temperature, which leads to premature snow thawing, increased evaporation, and thus earlier ground exposure and makes the ground dry in spring, thus promoting the spread of fire^34^. We believe that the methane emissions from the gradual thawing of permafrost may further improve the occurrence frequency, burning degree and spread area of this kind of wildfire. On the one hand, the "greenhouse effect" caused by the release of methane gas will increase the air temperature, which creates favorable conditions for wildfires. On the other hand, the combustibility of methane may also promote regional wildfires.

**Figure 12:**
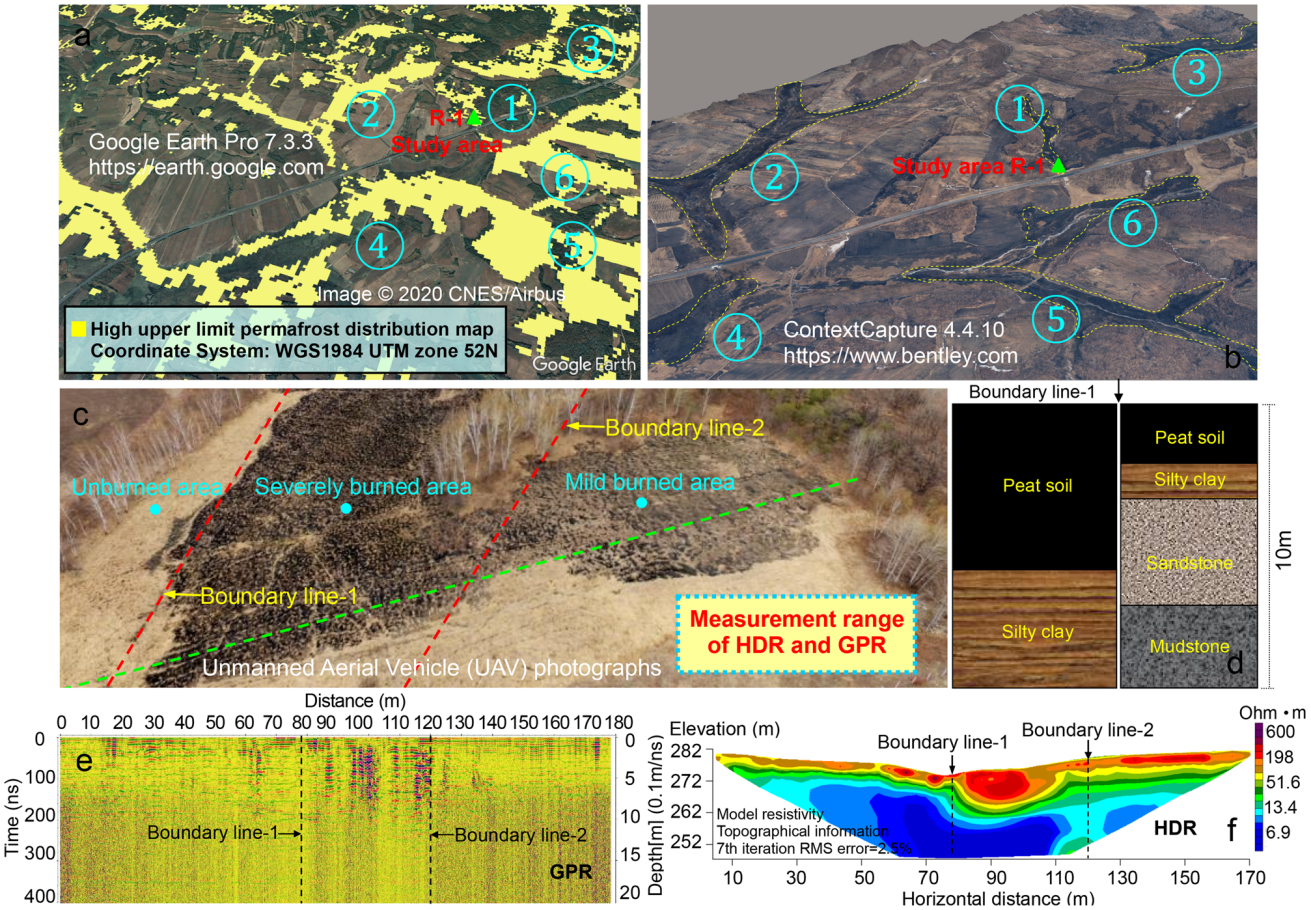
Wildfire situations in the study area R-1 (49°30′52″N, 127°18′21″E). (**a**) Distribution of permafrost near study area R-1, where the yellow area is the permafrost distribution area; the data come from Wang et al.^35^, Google Earth Pro 7.3.3 https://earth.google.com, (**b**) Distribution of wildfires near the study area R-1 in late March 2018, where the numbers ①–⑥ are the wildfire occurrence areas, ContextCapture 4.4.10 https://www.bentley.com, (**c**) Burned area of wildfire in the study area R-1 on March 25, 2018; the red dotted line is the boundary between the unburned area, the severely burned area and the mild burned area, and the green dotted line is the high-density electrical method (HDR) and the ground penetrating radar (GPR) survey line, unmanned aerial vehicle (UAV) photographs, (**d**) Soil sample components on both sides of dividing line 1, (**e**) Ground penetrating radar (GPR) detection result graph, (**f**) High-density electrical method (HDR) detection result graph.

Years of observation and discovery in the study area show the following (Fig. 12a): During the sudden increase in methane concentration in spring each year, there are multiple large-scale wildfires (①–⑥) in and near the study area (Fig. 12b). The areas where wildfires occur are concentrated mostly in low valley areas, which are consistent with the distribution of permafrost, and there are differences in the burned degree in the same area. It can be seen from the burned area of study area R-1 that under the condition that the combustible materials are evenly distributed and combustion conditions are consistent in the area, the burned degree of the study area is divided into three areas: an unburned area, severely burned area, and mild burned area (Fig. 12c).

To investigate the distribution and thickness of permafrost and the possible storage of methane hydrate in the study area without destroying the field environment, we used a high-density electrical method (HDR) and ground penetrating radar (GPR) to interrogate the field repeatedly (Fig. 12e,f). Due to the difference in the ice-water phase state, permafrost has the characteristic of high resistivity; that is, the resistivity of frozen soil is significantly higher than that of nonfrozen soil, so the types of permafrost can be divided according to the resistivity value obtained by high-density electrical method (HDR) inversion. Study shows that except for the frozen layer, the different microscopic distribution patterns of methane hydrate in permafrost, and the changes in the content of methane gas, pore water, and methane hydrate during the decomposition of methane hydrate will all cause changes in resistivity. The electrical resistivity of methane hydrate and gas is much greater than that of water. When the microscopic distribution of methane hydrate in the pores of the porous medium is low, the hindrance of the hydrate on the pore water communication cross-section is small, and the resistivity is relatively small. However, when the microscopic distribution of methane hydrate in the pores of the porous medium is more prevalent, the hindrance of hydrate on the pore water communication cross-section is greater, and the resistivity is larger^71–73^, so the permafrost layer has a relatively large resistivity and an uneven relatively high resistivity area affected by methane hydrate and methane gas. In addition, the electromagnetic wave attenuation in the frozen soil layer is very small, which will produce a good electromagnetic wave reflection surface at its upper and lower boundaries, which provides good geophysical conditions for the use of ground penetrating radar for frozen soil surveys^74,75^.

The drilling results show that the high and uneven resistivity areas obtained by high-density resistivity (HDR) and ground penetrating radar (GPR) are consistent with the permafrost and methane hydrate distribution areas obtained by field drilling. Based on field drilling, the permafrost layer in the burned area has a high ice content, and there is a fault within the unburned area due to geological movement (Fig. 12d), so the methane hydrates found in the permafrost layer in the study area may contain both metastable methane hydrates enclosed in shallow ice and deep stable methane hydrates due to geological movement. In addition, we observed that the green plants in the burned area grew worse than the green plants in the unburned area during the plant growth season each year, which may come from the reduction in soil oxygen content caused by the accumulation of high-concentration methane gas^76^. By combining the above results and comparing the burned area in the study area, it was found that the surface wildfire burned degree and range were positively correlated with the distribution characteristics of the permafrost, the thickness of the frozen soil, the content of methane hydrate and the concentration of surface methane; that is, the area with a larger frozen soil thickness and methane hydrate reserves and higher surface methane concentration is burned more heavily. It can be seen from Fig. 7c that the snow gradually thaws and the atmospheric humidity is relatively low, which further accelerates the emissions of methane gas and wildfires in spring. In addition, by combining Figs. 7d and 9c, it is found that the maximum value of surface methane concentration in the permafrost area occurs in spring, which is consistent with the highest monthly fire in southeastern Siberia in spring^32,33^. The special methane emission rules in the first (March–May) and third (September–November) stages are consistent with the seasonal fire changes in southeastern Siberia that reach local peaks in spring and autumn, and the peak in spring is approximately four times the peak in autumn^34^.

**Figure 13:**
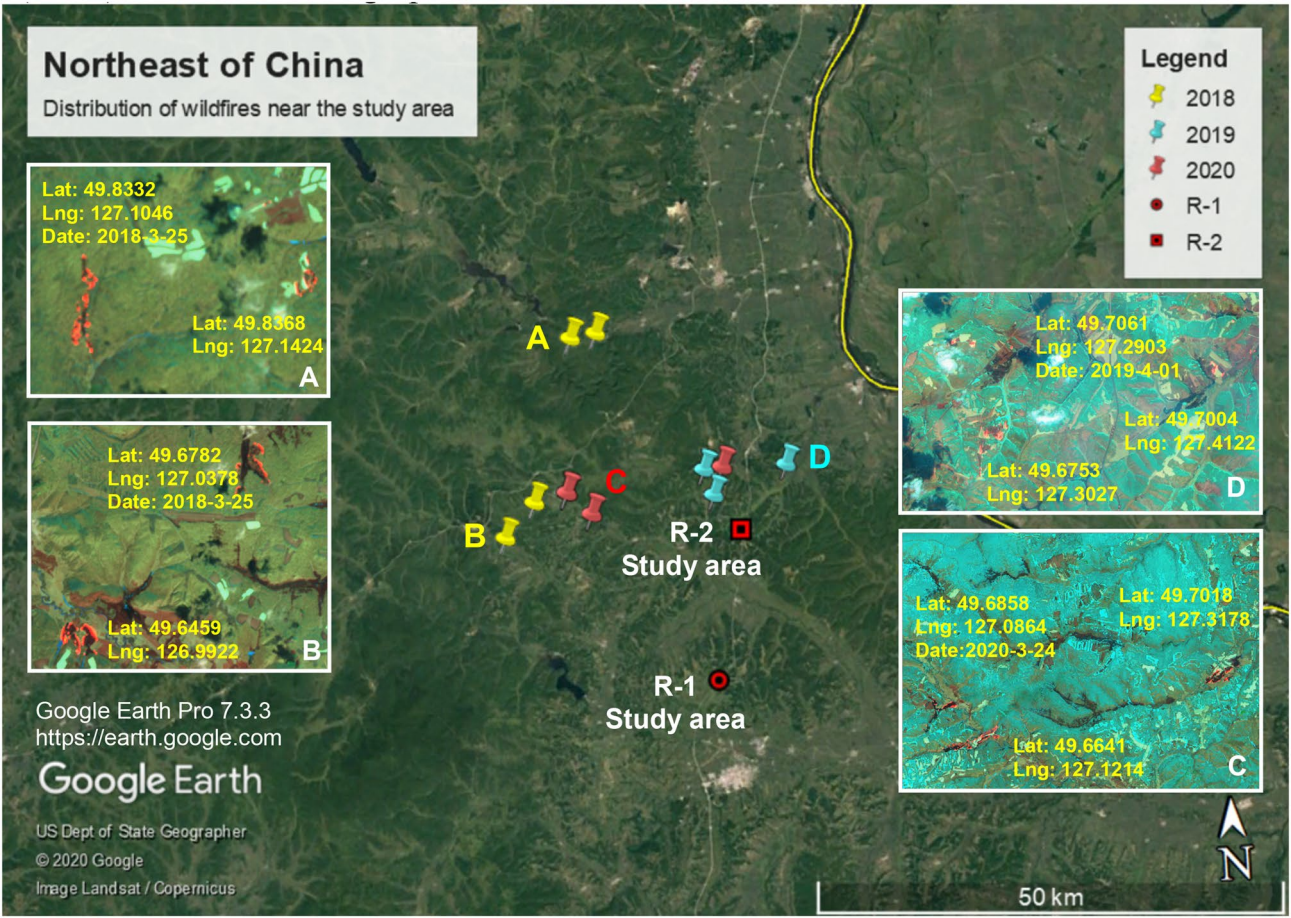
Distribution of large area wildfires in the study area. While excluding artificial fire points near farmland, fire points with larger burning ranges near the study area were selected in 2018–2020, EO-Browser 02 satellite image https://apps.sentinel-hub.com, Google Earth Pro 7.3.3 https://earth.google.com.

In addition, we conducted many years of monitoring of wildfires near the study area and found that excluding artificial fire points, wildfires occur mostly in low-lying areas such as valleys, which is consistent with the distribution of permafrost. The occurrence time of wildfire is concentrated; that is, the fire points in spring are concentrated mainly in the middle and late March, and the wildfire occurrence area has short-term nonrepeatability, that is, after a certain part burns, there will be no wildfire in the next year or several years again, but other similar parts in the area will have wildfires (Fig. 13). This phenomenon may be caused by the rapid consumption of high-concentration methane gas due to its own combustion, the destruction of permafrost and its internal microbial environment, although the combustion mechanism of methane gas in this study area is not very clear.

**Figure 14:**
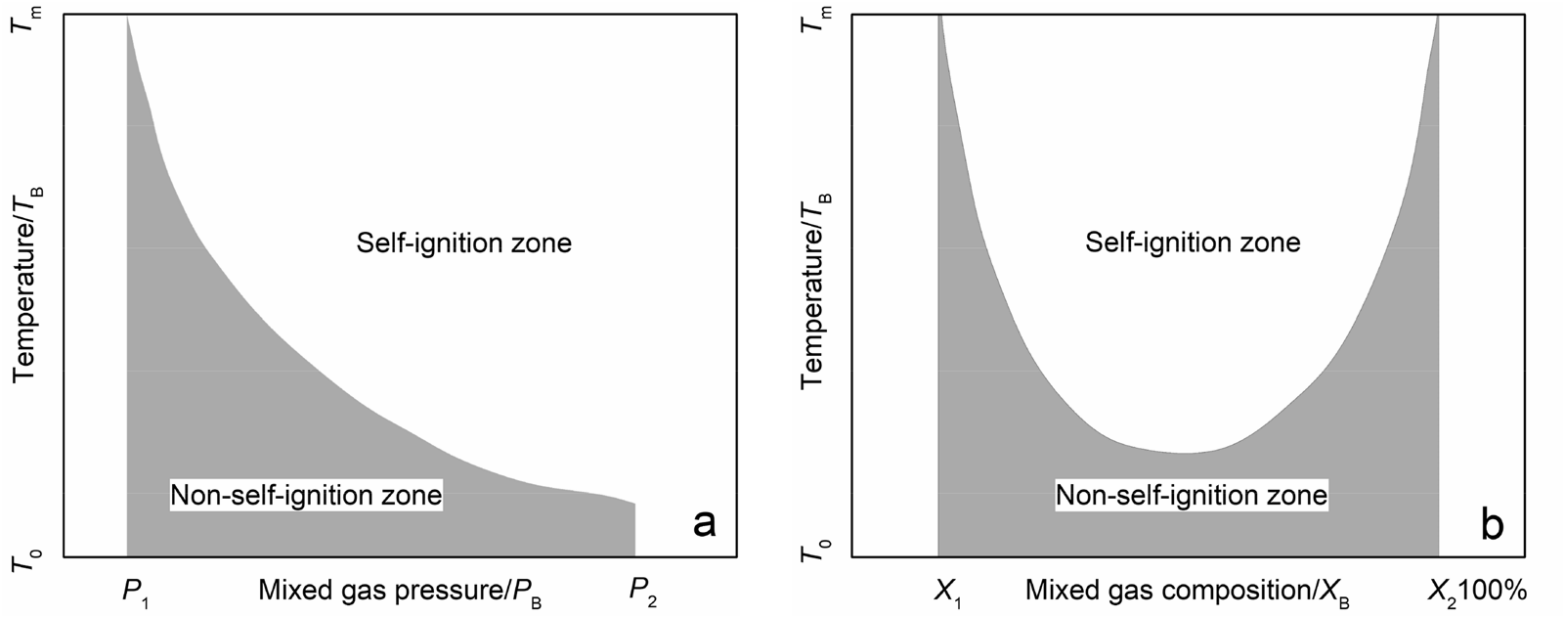
Thermal spontaneous combustion limit diagram. (**a**) Thermal spontaneous combustion temperature curve as a function of gas pressure, (**b**) Thermal spontaneous combustion temperature as a function of mixed gas content.

Study shows that under ideal conditions, 1 m^3^ of methane gas hydrate can decompose 164 m^3^ of methane gas^13^. With the gradual degradation of permafrost, the metastable methane hydrates stored in the permafrost ice layer and the deep stable methane hydrates gradually decompose. Driven by the pressure gradient, methane gas will migrate to the surface and accumulate under the frozen layer with the methane gas generated by the action of microorganisms, leading to the formation of a high-concentration, high-pressure methane atmosphere in the soil layer below the frozen surface. When the frozen layer thaws, the accumulated methane gas will be discharged into the surface. Combined with the thermal spontaneous combustion limit equation^61^, higher mixed gas pressure and methane gas content will reduce the thermal spontaneous combustion temperature of methane gas within a certain range, thereby promoting the thermal spontaneous combustion of methane gas (Fig. 14) Eqs. (2 and 3)^77^.

2 $$ \frac{{VQk_{0} n^{v} E}}{{RT_{0}^{2} aS}}e^{{ - \frac{E}{{RT_{0} }}}} = 1 $$

3 $$ \ln \frac{p}{{T_{0}^{(1 + 2/v)} }} = \frac{A}{{T_{0} }} + B $$

The equation of thermal spontaneous combustion is Eq. (2), where *α*, *S*, and *V* are the heat dissipation conditions of the system and *E*, *Q*, *k*_0_, and *v* are the chemical performance parameters of the mixed gas. When the heat dissipation conditions (*α*, *S*, *V*) and the chemical performance parameters (*E*, *Q*, *k*_0_, *v*) of the mixed gas are known in the system, under the given mixed gas temperature *T*_0_, the relationship between gas pressure and spontaneous combustion temperature can be obtained Eq. (3).

In addition, when methane gas is combined with aerosols (such as dust, sea salt, sulfate and black carbon and other atmospheric particles), the methane has a greater warming potential. The impact of methane emissions is greater than the emissions used in the current carbon trading plan or the Kyoto Protocol. The International Panel on Climate Change (IPCC) and treaties such as the Kyoto Protocol hold that ton-for-ton, methane is 25 times more potent than carbon dioxide at warming the planet. Furthermore, the interaction with aerosols increases methane's relative global warming potential (GWP) to approximately 33 times^78–80^. Therefore, the presence of aerosols will further increase the influence of methane gas on the warming of the atmosphere, thereby increasing the occurrence of wildfires. However, in addition to the above discussion, the influence of methane gas on the frequency of wildfires may be multifactorial, which will require further exploration.

**Figure 15:**
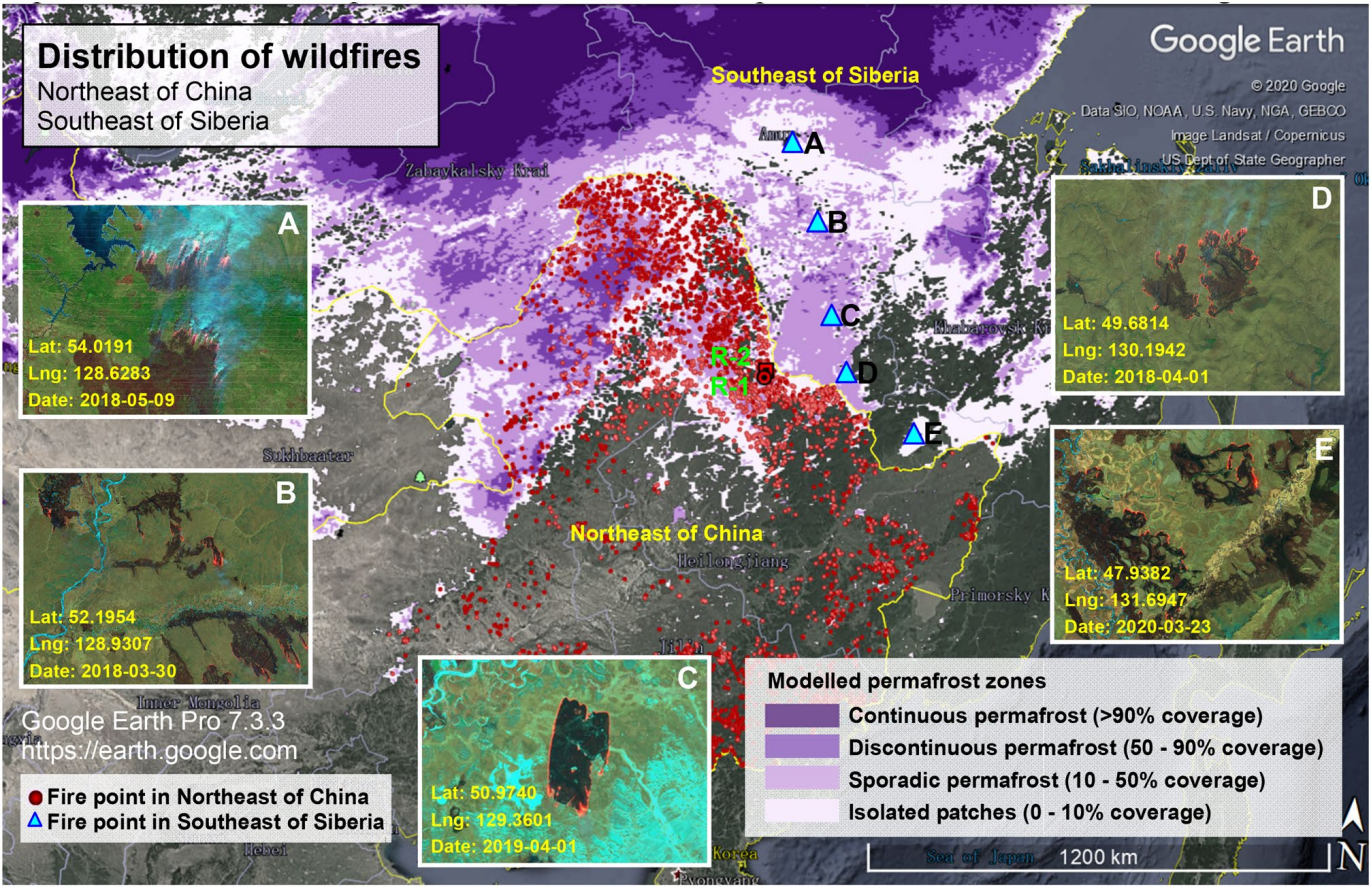
Wildfires near Northeast China and the Breya Mountains in Southeast Siberia. For Northeast China, the 2000–2018 fire point locations are obtained by the relevant fire management department. For Southeast Siberia, the 2018–2020 wildfire image is obtained through Sentinel 2. This area is located in the permafrost area in southeast Siberia and borders the study area, EO-Browser 02 satellite image https://apps.sentinel-hub.com, Google Earth Pro 7.3.3 https://earth.google.com.

It can be seen from the accumulated wildfire location in Northeast China in 2000–2018 obtained by the fire management department that the location of wildfires in this region is mainly in the permafrost distribution area, while the fire point distribution in the permafrost degradation area represented by the study area (Sunwu-Jiayin Basin in Northeast China) is more intensive than that in the continuous permafrost area (Fig. 15). Further, the Sentinel 2 (Sentinel-2 L1C) satellite image (Fig. 15) is used to conduct a statistical analysis of the 2018–2020 wildfire distribution in the permafrost area in southeastern Siberia bordering the study area: in the spring of each year (March–May), in addition to the Sunwu-Jiayin Basin study area in northeastern China, there are also large-scale wildfires in the southwest (Breya-Jieya Basin) and the southeast of Breya Mountain in Russia across the Heilongjiang River from the study area, and this region is a sedimentary basin with conditions for the formation and storage of methane hydrates. In addition, this region is located in the southern boundary of the Eurasian permafrost zone in a permafrost degradation zone, and the permafrost layer is relatively unstable.

### Establishment of a conceptual model

**Figure 16:**
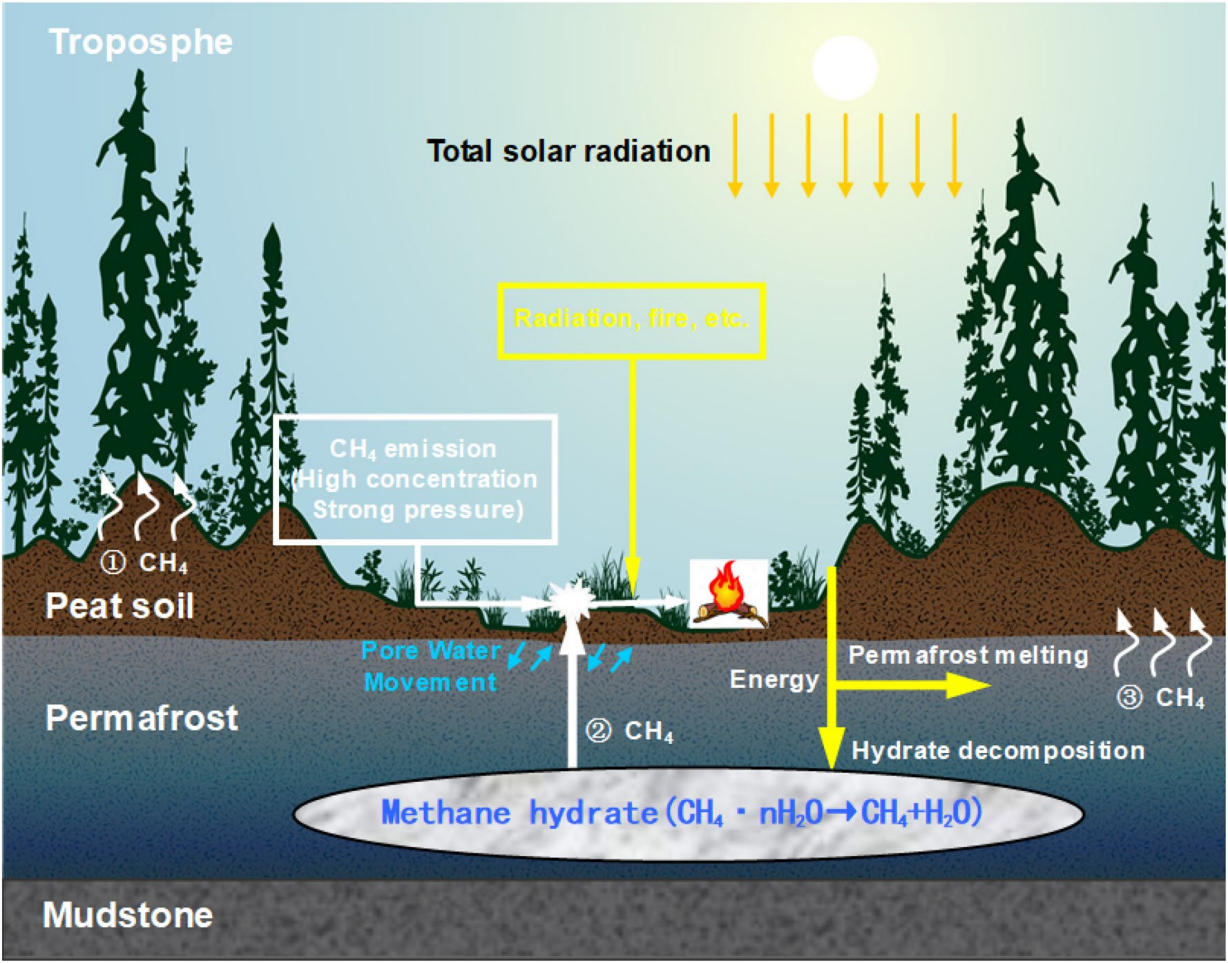
Conceptual model of methane emissions and its impact on wildfires in permafrost^81^. The top of the soil layer is peat soil with a certain thickness, the bottom is a permafrost layer and contains a certain amount of methane hydrate, and the bottom layer is mudstone.

Based on the above study, a conceptual model of methane emissions and their impact on wildfires in permafrost regions is established (Fig. 16). Among the relevant factors, the soil structure at different depths can be simplified from top to bottom: thicker peat soil, permafrost layer (mainly gravel sand clay, etc.), gravel-bearing mudstone layer, and certain reserves of methane hydrate in the permafrost layer; the distribution is relatively uneven. The permafrost layer is gradually degraded, and the methane hydrate stored in it will be disturbed to decompose to form a high concentration of methane gas. This part of the gas will gradually move to the surface under the effect of a pressure gradient, so the main sources of surface methane gas in the study area include microbial action and decomposition of methane hydrate in permafrost. In addition, the methane gas accumulated under the frozen layer in spring exhibits high pressure and high concentration during the surface emission process. It will be highly likely to burn under the influence of external conditions (solar radiation, fire sources, etc.), and then spread to form large-scale wildfires and forest fires.

## Discussion

Due to the influence of environmental factors, the thickness of permafrost in the northern Xiao Xing'an Mountains in Northeast China is gradually decreasing. Organic matter and methane hydrate stored in the permafrost gradually enter the atmosphere in the form of methane gas. Methane emissions show seasonal change, and the annual methane emissions are mainly divided into three stages. The annual maximum surface methane concentration occurs from March to May each year (high-concentration short-term emission stage). The methane gas generated by the gradually decompose of metastable methane hydrates stored in the permafrost ice layer and the deep stable methane hydrates, and the action of microorganisms may be the main source of high-concentration methane gas entering the surface at this stage. Due to the extremely high energy density of methane hydrate, a high-concentration, high-pressure methane atmosphere will be formed in the soil layer under the freezing surface under the combined driving of a pressure gradient and frozen soil temperature rise, and the high-concentration, high-pressure conditions will reduce the thermal autoignition temperature to a certain extent. When methane gas is combined with aerosols (such as dust, sea salt, sulfate and black carbon and other atmospheric particles), the methane has a greater warming potential. Therefore, this methane gas may increase the risk of wildfires in permafrost degraded areas by its spontaneous combustion and increase the regional atmospheric temperature. Since the occurrence of wildfires will cause a large amount of carbon and heat emissions, the greenhouse effect of this methane gas, by promoting the occurrence of wildfires, may be much higher than its own impact on the greenhouse effect. In addition, the permafrost will thaw further after the forest fire, and the respiration rate of microorganisms after the fire is three times that before the fire, which greatly increases the diffusion of CO_2_, CH_4_ and N_2_O in the atmosphere^82^. Moreover, the fire affects the water and heat conditions of the permafrost area. After the fire, the surface albedo is reduced, the surface heat condition is changed, the thickness of the active layer is deepened, and the loss of organic carbon (SOC) and nutrients accelerated. After the fire, the whole forest system needs to recover from the disturbance for more than a century^83^.

However, study on the formation, transmission, restraint mechanism and control factors of methane in permafrost regions is still relatively weak. We still lack a quantitative understanding of the role of each process in overall methane emissions, and the scope of study is mostly regional and offers less understanding of methane gas emission mechanisms and emissions from the decomposition of methane hydrates in permafrost regions. Moreover, the current survey and study inputs for methane hydrates focus mainly on the energy direction, while the areas with less methane hydrate reserves or uneven distribution are basically not considered. At present, a large amount of methane hydrate reserves have been found under permafrost in the Mohe Basin in northern Northeast China^65^; except for the study area in this paper, the range of methane hydrate reserves in the stable zone is approximately 0.49 × 10^12^–0.79 × 10^12^ m^3^. However, due to the special geological structure and distribution, no large-scale mining has been carried out to date, and there are many areas in the permafrost area, such as the Mohe Basin, that contain methane hydrate but are difficult to mine or are unexplored. With the southern boundary of the permafrost zone moving northward in Eurasia, earthquake and the seasonal changes in water content, this part of the methane hydrate will gradually decompose to form methane gas and enter the atmosphere. Therefore, there is still great uncertainty in this carbon transfer due to the high speculation of methane hydrate quantity estimation, the complexity of the hydrate stability control mechanism, and the relatively rough atmospheric methane concentration remote sensing image and climate model.

To achieve overall monitoring of methane gas emissions and their impact on wildfires in the permafrost area, the primary task is to increase the number of meteorological stations to form a monitoring network. To date, our study team has added several meteorological stations near the study area and will rely on Field scientific observation and research station of the Ministry of Education—Geological environment system of permafrost area in Northeast China (FSSE-PFNEC) to set up monitoring stations in the more northern Tahe and Mohe areas and gradually expand their number as learning progresses. In addition to the basic observation parameters of methane concentration, air temperature, pore water pressure and soil temperature, data monitoring such as air humidity, soil humidity, solar radiation, rain, snow and soil heat flux etc. are added. In addition, our study team will install monitoring devices in the study area to monitor the field conditions in real time to better understand the cause, time and location of wildfires. However, the installation and maintenance of the monitoring network is a challenging problem. Since the monitoring environment is in the permafrost region, the temperature is low and affected by environmental factors such as snow. Therefore, the instrument must have high practicability and durability, and the accuracy of the instruments must be regularly corrected, so the study team has trained a number of professional staff.

A series of environmental problems caused by global warming, such as fires in cold regions, will not only further aggravate global climate change but also affect local human activities and forest resources, so scientists from all over the world need to work together and cooperate with relevant geological departments to comprehensively explore the distribution of methane hydrate in permafrost regions and carry out monitoring site layouts. In this way, the known area can be treated in advance to reduce the possibility of wildfires and protect the atmospheric environment and forest resources according to the possible loss degree.

## Methods

### Meteorological station observations

This paper adopts the meteorological monitoring system (http://www.qixiangshuju.com) and the ground soil physical parameter monitoring system (http://www.hzjly.cn) to monitor the meteorological data in real time on site. In January 2009, a monitoring system was set up in study area R-1 (49°30′52″N, 127°18′21″E, altitude 278 m), and in January 2019, a monitoring system was set up in study area R-2 (49°39′28″N, 127°21′5″E, altitude 229 m). The meteorological station model is GD24-YCXQ, which is mainly composed of a data acquisition host, data analysis system, data transmission system, Internet of Things sensors, GPRS wireless transmission system, solar power supply system, etc. As the value of the meteorological element changes, the output power of each sensor element also changes, and the data collector controlled by the CPU collects data in real time. After linearization and quantification processing, the conversion from process quantity to element quantity is realized, and then the data are filtered to obtain the value of each meteorological element. The ambient temperature of the meteorological station is − 30–70 °C, and 64 GB of data are stored every hour. This system mainly includes a surface methane concentration sensor (QT21-BX80-CH_4_) and an air temperature sensor (GD51-KWSY). For the methane concentration sensors (QT21-BX80-CH_4_), the measurement range is 0–1000 ppm, the measurement accuracy is ± 1% (F.S.), the resolution is 0.1 ppm, and the temperature range is − 25–45 °C. For the air temperature sensors (GD51-KWSY), the measurement range is − 30–120 °C, the measurement accuracy is ± 0.2 °C, the resolution is 0.1 °C, and the working temperature range is − 40–75 °C. The ground soil physical parameter monitoring system uses a soil pore water pressure sensor (KXR-3034) and a soil temperature sensor (HJ6-FM-TWB) to monitor related parameters. The soil pore water pressure sensor (KXR-3034) is a vibrating wire pore water pressure sensor; the measurement range is − 1.0–1.0 MPa, the measurement accuracy is ± 0.05% (F.S.), the resolution is 0.01 kPa, and the temperature range is − 25–60 °C. The pore water pressure sensor uses a 4–20 mA output, and the output current signal can be converted into the actual pore water pressure value. The KXR-3034 vibrating wire pore water pressure calculation equation is shown in (4). where *P* is the pressure value of the measured pore water pressure sensor (MPa), *K* is the sensitivity coefficient (MPa/Hz^2^, the values are 1.229, 1.181, 1.001), *f*_0_ is the initial frequency value (the values are 1684.3, 1436.3, and 1207.6), and *f*_i_ is the working frequency value.

4 $$ P = K\left( {f_{i}^{2} - f_{0}^{2} } \right) $$

The soil temperature sensor (HJ6-FM-TWB) has a measurement range of − 30–120 °C, a measurement accuracy of ± 0.2 °C, a resolution of 0.1 °C, and a working temperature range of − 40–75 °C. In addition to the methane concentration sensor, we also used the SKZ1050-CH_4_ handheld methane detector to detect the methane concentration on the surface of the study area. The measurement range is 0–100% Lower Explosive Limited (LEL), the measurement accuracy is ± 2% (F.S.), and the resolution is 0.1% LEL.

### High density resistivity (HDR)

This paper uses the WGMD-9 super high-density electrical method system to conduct multiple detections of the permafrost distribution in the study area R-1 (49°30′52″N, 127°18′21″E). Because of the different types of frozen soil, the dynamic changes in frozen soil will have a prominent impact on the physical properties of geological bodies. At the freezing point, the phase change from conductive water to nonconductive ice increases the resistivity significantly, so the permafrost can be explored through different resistivities. The operating temperature of this system is − 10–60 °C, the storage temperature is − 20–60 °C, and the electrode spacing is 3 m. The data processing adopts the quasi-Newton optimization nonlinear least squares algorithm, adjusts the model resistivity by the model correction amount, and reduces the difference between the actual apparent resistivity and calculated resistivity. The areas with a resistivity of 100 Ω·m and above in the study area are permafrost distribution areas. To ensure accuracy, we use both the Wiener and Schlumberger permutations to carry out multiple detections on September 27, 2010–2019.

### Ground penetrating radar (GPR)

In this paper, RIS-FastWave GPR produced in Italy was used to detect the permafrost distribution in study area R-1 (49°30′52″N, 127°18′21″E) on September 27, 2010–2019. The monitoring system is based on microwave interference, synthetic aperture and linear continuous wave technology, with no need to approach the target and no need to install sensors in the target area. The telemetry distance can reach 4 km, and the main engine frequency is a 17.05–17.35 GHz linear continuous wave. The system bandwidth is 200–300 MHz, the range resolution is 0.5 m, and the angular resolution is 4.5 mrad.

### Sentinel-2 L1C satellite image

In this paper, the satellite images (https://apps.sentinel-hub.com/eo-browser/) provided by Sentinel-2 L1C are used to track 2018–2020 wildfires in Northeast China and East Siberia. Sentinel-2 L1C is the atmospheric apparent reflectance product after orthorectification and subpixel geometric precision correction and is composed of UTM/WGS84 projection orthophoto images. The L1C product image is projected to cartographic coordinates using the digital elevation model (DEM) during orthorectification, which is the reflectance image of the top of atmosphere (TOA). In addition, the relevant information of the radiation measurement processed as each pixel of the L1C product is recorded. According to the resolution of different spectral bands, L1C products are resampled with fixed ground sampling distances (GSDs) of 10, 20 and 60 m.
